# Serving by local consensus in the public service location game

**DOI:** 10.1038/srep32502

**Published:** 2016-09-02

**Authors:** Yi-Fan Sun, Hai-Jun Zhou

**Affiliations:** 1 Center of Applied Statistics, School of Statistics, Renmin University of China, Zhong-Guan-Cun Street 59, Beijing 100872, China; 2 Key Laboratory of Theoretical Physics, Institute of Theoretical Physics, Chinese Academy of Sciences, Zhong-Guan-Cun East Road 55, Beijing 100190, China; 3 School of Physical Sciences, University of Chinese Academy of Sciences, Beijing 100049, China

## Abstract

We discuss the issue of distributed and cooperative decision-making in a network game of public service location. Each node of the network can decide to host a certain public service incurring in a construction cost and serving all the neighboring nodes and itself. A pure consumer node has to pay a tax, and the collected tax is evenly distributed to all the hosting nodes to remedy their construction costs. If all nodes make individual best-response decisions, the system gets trapped in an inefficient situation of high tax level. Here we introduce a decentralized local-consensus selection mechanism which requires nodes to recommend their neighbors of highest local impact as candidate servers, and a node may become a server only if all its non-server neighbors give their assent. We demonstrate that although this mechanism involves only information exchange among neighboring nodes, it leads to socially efficient solutions with tax level approaching the lowest possible value. Our results may help in understanding and improving collective problem-solving in various networked social and robotic systems.

The healthy functioning of a human society depends on various public services or facilities. Public services (e.g., schools, hospitals, parks, garbage disposal plants, …) are costly to construct and the costs are shared by the whole society through tax. On the other hand it is often the case that a service (say a hospital) located at one place will also serve the residents of neighboring places. By appropriately choosing the public service locations, the total construction cost to cover the whole society may be greatly reduced. This public service location task is an important and challenging issue faced by a modern human society and is an active research topic in the fields of algorithmic game theory^1^ and network game^2^,^3^,^4^.

Governmental institutions may prefer to solve such public service location problems in a top-down and centralized manner. A central planner will collect all the network information about the social system, and then it will take this structural knowledge as input to a global optimization algorithm to obtain a minimum-cost solution. Such a centralized approach, however, has serious drawbacks. Firstly, it requires a central planner and requires complete information about the network. Secondly, the vast members of the society are excluded from the decision-making process and their individual preferences are not necessarily incorporated. This lack of involvement may cause people to suspect that their interests are compromised, leading to strong friction and unwillingness. There are many recent large-scale events of such types of distrust and friction. For example in 2007 some residents in Xiamen (a major city of southeast China) protested against the settlement of a PX (paraxylene) plant and eventually forced its relocation^5^.

A completely different approach for solving the public service location problem is to let individual agents make decisions in best response to the choices of their neighboring agents. Under such a free-market mechanism, if an agent can access service from a neighboring agent, it will have no motivation to be a service provider itself, and an agent will choose to be a service provider only if none of its network neighbors offers service. This best-response mechanism has been investigated in the recent literature^2^,^3^,^4^,^6^,^7^, and it was found that the resulting maximal independent-set solutions are far from being socially efficient.

Given the failure of the best-response mechanism, how to facilitate cooperative decision-making among members of a society so that socially efficient solutions can be achieved without centralized planning? The present paper addresses this important theoretical question and offers a local-consensus selection mechanism for the public service location problem. This decentralized mechanism lies between the above-mentioned centralized and individualized mechanisms. Briefly speaking, the basic rules are that agents in need of service recommend their network neighbors of highest local impact (to be precisely defined later) as candidate service providers, and an agent may be chosen as a service provider only if all its non-server neighbors are happy with this appointment. This distributed selection mechanism does not require the global structural information of the system but only involves local-scale information exchange. Yet very encouragingly we find that it leads to socially efficient solutions with tax level approaching the lowest possible value.

Our theoretical results suggest that distributed decision-making through local consensus can be an efficient mechanism for solving the network public service location problem. This cooperation mechanism may also be useful for other network resource allocation problems^8^,^9^,^10^,^11^. In addition it may have potential applications in robotic swarm systems for collective problem-solving^12^ and may also be relevant to the research branch of distributed algorithmic mechanism design^13^,^14^.

## Public Service Location Game

**Figure 1:**
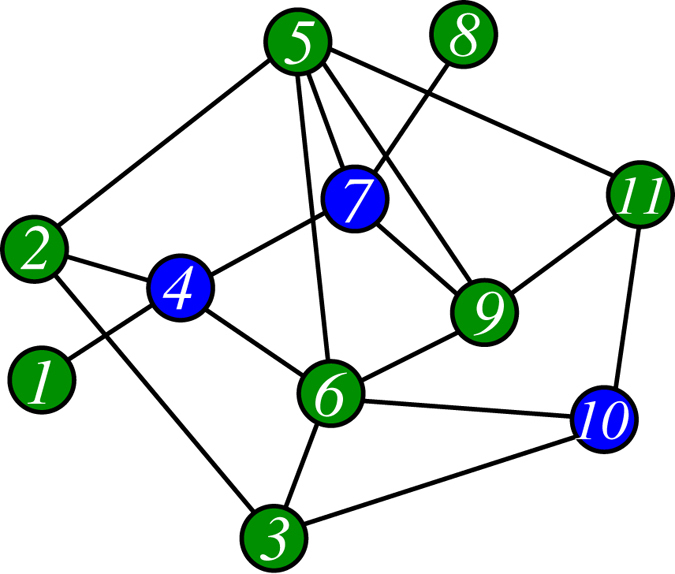
An illustration of the public service location problem. There are *N* = 11 nodes (agents) and *M* = 16 links. Nodes 4, 7, and 10 are servers and they pay the total service construction cost of *N*_1_ = 3. All the other eight nodes are pure consumers, they access service from neighboring servers. Each consumer pays a tax 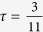 to reduce the net construction cost of each server back to the fair value 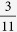.

Consider a society formed by *N* agents, each of which interacting with a set of neighboring agents. The neighborhood property is reciprocal so that if agent *i* is a neighbor of agent *j* then *j* is also a neighbor of *i*. Every agent is dependent on certain essential public service provided by itself or by its neighbors^2^,^4^. We assume that hosting this service incurs a construction cost, and without loss of generality we set this cost to be unity. The service provided by a hosting agent (referred to as a server) is not excludable but is accessible to all the neighboring agents (Fig. 1), so an agent does not need to be a server itself if at least one of its neighbors is already a server. This is called a property of strategic substitutes in the literature^2^,^3^. If the construction costs are borne only by the servers, naturally no agent will volunteer to be a server but will wait the neighboring agents to build the public service, leading to extortion and the “tragedy of the commons”. The only fair solution under this cost no-sharing rule will be that every agent hosts the service, which is not socially efficient.

In this paper, therefore, we assume that the agents have reached the agreement that free-riding is not allowed and that the total service construction cost is evenly shared by all the agents in the society. The public service location problem then is a network game^3^,^15^ in which each agent makes decision under the strong self-interest of having access to the service and the weak incentive of lowering the number of servers. We can represent the public service location problem by a network *G* of *N* nodes and *M* links. Each network node represents an agent and the link (*i*, *j*) between nodes *i* and *j* signifies that *i* can access the service produced by *j* and *j* can access the service produced by *i* (Fig. 1). The network structure is fixed in time. Each node *i* can choose to be a server (denoted by occupation state *c*_*i*_ = 1) or just be a consumer (state *c*_*i*_ = 0) and it might change between these two choices before a final solution is reached. A solution of this problem is an occupation configuration *c* ≡ (*c*_1_, *c*_2_, …, *c*_*N*_) such that each node is either a server (e.g., nodes 4 and 7 in Fig. 1) or is a consumer surrounded by one or more servers (e.g., nodes 2 and 6 in Fig. 1). The total construction cost is equal to the total number *N*_1_ of servers in the solution *c*, and 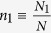 is the fraction of servers. Because of the fair-sharing rule, each consumer needs to pay a tax *τ* = *n*_1_ and each server will receive a subsidy (1−*τ*) so as to reduce its net construction cost back to τ.

## Methods

### Centralized planning

A central planner, assumed to have complete information about the network *G*, can try to get an optimal solution *c* for the public service location problem by global optimization. The set Γ of servers, with the property that every node in *G* either belongs to Γ or has a neighboring node in Γ, is nothing but a dominating node set^16^,^17^. An optimal service location solution *c* corresponds to a minimum-sized dominating set.

The minimum dominating set problem is a NP-hard (nondeterministic polynomial hard) combinatorial optimization problem. Although not rigorously proven, it is widely believed that a guaranteed optimal solution can only be obtained by checking an exponential number of candidate solutions. In practice one can solve this problem approximately, and so far the best way appears to be converting it to a spin glass model^18^ and then treating it by methods of statistical physics^19^. Such a spin glass approach offers an estimate about the size of minimum dominating sets, and it also offers a powerful message-passing algorithm called BPD (belief propagation-guided decimation, see Supplementary Information) for solving single network instances. For random networks, the solutions obtained by the BPD algorithm are very close to be true minimum dominating sets^18^. Here we take the result obtained by the BPD algorithm as a good proxy of the true optimal solutions for the public service location problem.

### Best-response mechanism

A simplest decision-making strategy is best response to the current situation of the neighborhood^2^,^7^,^20^. If node *i* has one or more neighboring servers it just chooses to be a consumer (*c*_*i*_ = 0), otherwise it chooses to be a server (*c*_*i*_ = 1). Nodes in the network update their choices non-synchronously until all are satisfied with their last choice. After a transient period of choice changes, this best-response dynamics converges to a solution *c* in which all the servers are separated from each other and every consumer has at least one neighboring server. The set of servers therefore is a maximal independent set of the network^6^. By definition a maximal independent set is a vertex set *S* with two properties: first, there is no link between any two nodes of this set; and second, every node not belonging to *S* must have at least one nearest neighbor in *S* (namely, *S* must be a dominating set of the network *G*).

### Local-consensus mechanism

**Figure 2:**
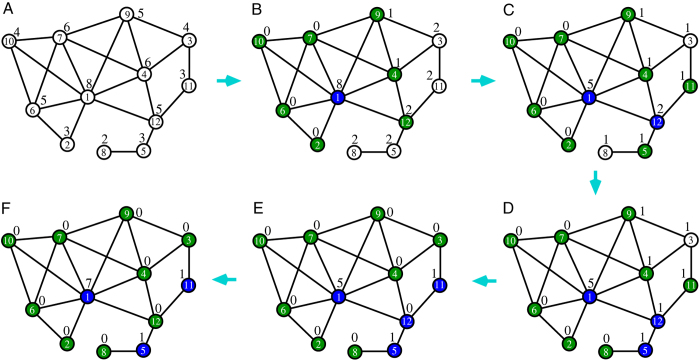
The local-consensus mechanism for solving the public service location problem. Blue nodes are servers, green nodes are served consumers, and white nodes are unserved consumers. The non-negative integer beside a node is its impact value. (**A**) Initially there is no server, and node 1 is the only candidate server who is agreed by all its neighbors. (**B**) After node 1 changes to be a server, all its neighbors are served, and then {3, 5, 8, 11, 12} becomes the set of server candidates. (**D**,**E**) Nodes 12, 5 and 11 are then sequentially chosen as servers, leading to a solution with node 12 having zero impact. (**F**) Node 12 is changed back to be a consumer, resulting in the final server set {1, 5, 11} which is an optimal solution.

The impact *f*_*i*_ of a node *i* in the network is defined as follows: If *i* is a server (*c*_*i*_ = 1), *f*_*i*_ is the total number of nodes which rely exclusively on *i* to get service (e.g., node 1 has impact *f*_1_ = 8 in Fig. 2B); if *i* is a consumer (*c*_*i*_ = 0), its impact is the increase in the number of served nodes if *i* becomes a server (e.g., node 12 has impact *f*_12_ = 2 in Fig. 2B). The impact of a node might change with time before a final solution *c* is adopted.

We now propose a local-consensus selection mechanism to reach a cooperative solution. We assume that every node can access the latest impact values and occupation states of all its neighbors. The servers for the network are assigned sequentially until every node is being served. Initially there is no server in the network and the impact of a node *i* is simply *f*_*i*_ = 1 + *d*_*i*_, where the degree *d*_*i*_ is the number of this node’s neighbors (Fig. 2A). At each elementary time interval every non-server node *i* checks its neighborhood: if *i* is *unserved*, then it regards a neighboring node *j* as suitable to be a server if and only if *j* has the highest impact among *i*'s neighboring nodes and *f*_*j*_ is no less than *f*_*i*_; if node *i* is *served*, then it regards a neighboring unserved node *k* as suitable to be a server if *f*_*k*_ ≥ *f*_*i*_. An unserved node becomes a server candidate if it is recommended as a suitable server by all its neighbors (e.g., node 1 in Fig. 2A). A served non-server node also becomes a server candidate if all the neighboring unserved nodes recommend it (e.g., node 12 in Fig. 2B). A randomly chosen node (say *k*) out of all the server candidates is then appointed as a server (*c*_*k*_ = 1), while all its neighbors update their impacts and the game process repeats (see Fig. 2B–E). After all the servers are appointed through such a local-consensus mechanism, if a server has zero impact (e.g., node 12 in Fig. 2E), then it is changed back to be a consumer. This polish process is carried out in a random sequential manner until all the remaining servers have positive impact (Fig. 2F).

### Local-share mechanism

Another distributed selection mechanism we examine is the local-share mechanism. Its key difference with the local-consensus mechanism is that the cost of building a public service is only shared locally. The servers are also assigned successively in the local-share mechanism (see Fig. S1 in Supplementary Information for an illustration). Initially, all the nodes are unserved. In each elementary step, every unserved node *i* checks its unserved neighbors, and it considers an adjacent node *j* suitable to be a server if and only if its impact *f*_*j*_ is the highest among all the unserved neighbors and *f*_*j*_ ≥ *f*_*i*_. An unserved node becomes a server candidate if it is endorsed by all its unserved neighbors, and one of such candidate nodes (say *k*) is then randomly chosen and appointed as a server. All the initially unserved neighbors of node *k* will then get service from *k* and these nodes share the construction cost with server *k* in a fair manner. The server selection process keeps going on until all the nodes are served. It is easy to see that the final set of server nodes is a maximal independent set of the network.

## Results

We first consider the public service location problem on random networks of mean node degree *c*. Four different types of random networks are investigated here: Erdös-Rényi (ER), regular random (RR), exponential (EX), and scale-free (SF). The node degrees of a large ER network obey the Poisson distribution with mean value *c*, so that the probability *P*(*d*) of a randomly chosen node to have *d* attached links is 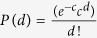. The node degrees of an EX network obey the exponential distribution with mean value *c*, so 
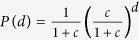
. Every node in a RR network has the same integer degree *K* (so the mean degree *c* = *K*). These three types of random networks are homogeneous in the sense that the degrees of all the nodes do not deviate much from the mean value *c*. On the other hand, a SF network is very heterogeneous and the degree distribution *P*(*d*) decays slowly in a power-law form *P*(*d*) ∝ *d*^−*γ*^ with exponent *γ* > 1 (we set *γ* = 3 in this work). Scale-free random networks are better models of real-world networked systems than ER or the other two types of homogeneous random networks^21^.

We apply the three different decentralized server selection mechanisms (best-response, local-share, local-consensus) to the same sets of random network instances and compare the obtained solutions with the solutions obtained by centralized planning (the global BPD algorithm).

**Figure 3:**
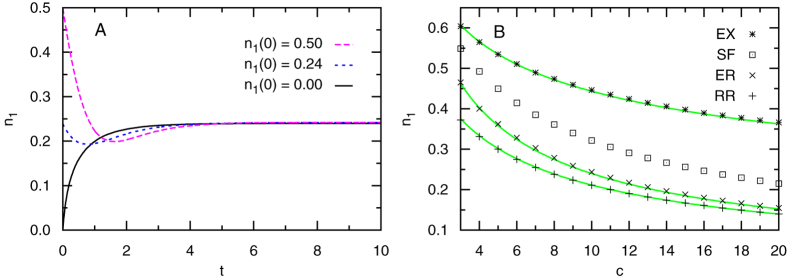
Results obtained by the best-response mechanism. (**A**) The fraction *n*_1_ of servers in an ER random network of *N* = 10^5^ nodes and mean degree *c* = 10 during the best-response dynamics. At each time interval *δt* = 1/*N* a node *i* is chosen uniformly at random from the network and its occupation state (*c*_*i*_ = 1 or *c*_*i*_ = 0) is updated. The three curves correspond to three different initial conditions with fraction of servers being 0.5, 0.24 and 0.0, respectively. (**B**) The final fraction *n*_1_ of servers obtained for four types of random networks of mean degree *c*: regular random (RR, pluses), Erdös-Rényi (ER, crosses), scale-free generated through the static model^29^ with decay exponent *γ* = 3.0 (SF, squares), and exponential (EX, stars). The three solid lines are the theoretical predictions (see Supplementary Information). We do not draw the theoretical curve for SF networks due to the numerical difficulty of integrating Eq. (S14).

For the best-response mechanism, we show in Fig. 3A the evolution of the fraction *n*_1_ of serves in a single ER random network with *N* = 10^5^ nodes and *M* = 5 × 10^5^ links (on average each node has *c* = 10 neighbors). The final value of *n*_1_ ≈ 0.240 is in excellent agreement with the predicted value of *n*_1_ ≈ 0.2398 by a mean field theory^6^. In general, the final fraction of servers reached by the best-response mechanism is *n*_1_ = ln(1 + *c*)/*c* for an ER network of mean degree *c* (Fig. 3B).

The same quick converging behavior is observed for many other random network instances and real-world network instances. For RR networks of degree *K*, the prediction^22^ is that *n*_1_ = [1 − (*K* − 1)^2/(2−*K*)^]/2, which is confirmed by simulations (Fig. 3B). For EX random networks the prediction is *n*_1_ = [(1 + 3*c*)^2/3^ − 1]/(2*c*) (see Eq. (S24) of Supplementary Information), which is again confirmed by simulations (Fig. 3B). The simulation results on SF networks are also shown in Fig. 3B. The mean field theory [Eqs (S14) and (S15) of the Supplementary Information] is also applicable to random SF networks but the numerical results are not shown due to difficulty of integrating Eq. (S14).

**Figure 4:**
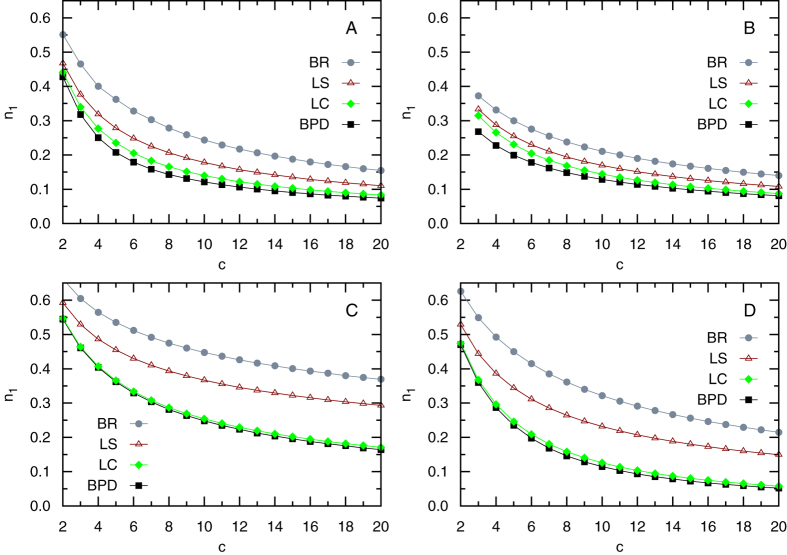
The fractions *n*_1_ of servers in solutions obtained by the best-response mechanism (BR, circles), the local-share mechanism (LS, triangles), the local-consensus mechanism (LC, diamonds), and the global BPD algorithm (squares). Each data point is the averaged result over 64 network instances with *N* = 10^5^ nodes and mean degree *c*. We consider four types of random networks: ER (**A**), RR (**B**), EX (**C**), and SF with decay exponent *γ* = 3.0 generated through the static model^29^ (**D**). The solid lines are just guide to the eye.

Figure 4 summarizes our systematic comparative results on the different mechanisms. For all the four types of examined random networks, we find that the best-response mechanism always has the worst performance; the local-share mechanism performs a little bit better but the fractions *n*_1_ of needed servers are still much higher than the minimum values. In contrast, the solutions obtained by the local-consensus mechanism are always very close to the BPD solutions in terms of the server fraction *n*_1_, indicating that this mechanism is able to reach nearly optimal solutions for the public service location problem. Especially interesting to notice is that the local-consensus mechanism works almost perfectly for SF random networks, which resemble real-world complex systems in terms of structural heterogeneity.

Next we compare the performances of the three decentralized selection mechanisms on a set of real-world social and infrastructure network instances and summarize the simulation results in Table 1. Again we find that the best-response mechanism has the worst performance, the local-share mechanism performs slightly better but it is still far from being satisfactory. In contrast, we find that the local-consensus mechanism performs almost equally well as the global BPD algorithm for all these examined network instances. It seems that the strong local structural correlations of real-world networks do not hinder the performance of the local-consensus mechanism.

Compared with the central planning approach, a nice advantage of the local-consensus mechanism is that each node does not need to know the structure of the whole network *G* but only needs to know who are the neighbors and what are their states (server, unserved or served consumer) and current impact values. The essence of this decentralized mechanism is that non-server nodes recommend their highest-impact neighbors as candidate servers. An unserved node will only be selected as a server if it currently has the highest impact among its neighbors and the neighbors of its unserved neighbors. Through this mechanism, a served consumer may change to be a server in response to the recommendation of all its unserved neighbors.

The results of Fig. 4 and Table 1 clearly demonstrate that the local-consensus mechanism is superior to the local-share mechanism in terms of total service construction cost. Another advantage of the local-consensus mechanism is that every node contributes equally to the total construction cost. This property of global equality is not guaranteed by the local-share mechanism. Indeed we find that the local-share mechanism leads to considerable inequality among the nodes (Fig. S2 in Supplementary Information): Some fortunate nodes need only to pay very little while the majority of the nodes need to pay considerably more for the construction cost. This inequality situation persists even when all the nodes of the network have the same degree (see Fig. S2B), suggesting that it is an intrinsic shortcoming of the local-share mechanism.

**Figure 5:**
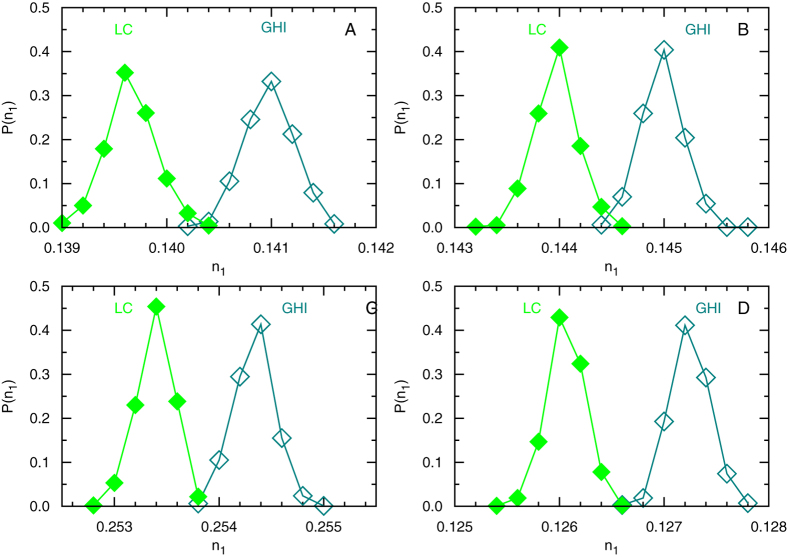
Comparing the performances of the local-consensus selection mechanism (LC, filled diammonds) and the greedy highest-impact algorithm^16^,^23^,^24^ (GHI, unfilled diammonds) on four types of random networks: ER (**A**), RR (**B**), EX (**C**), and SF with decay exponent *γ* = 3.0 generated through the static model^29^ (**D**). Each histogram *P*(*n*_1_) of server fraction *n*_1_ is constructed by sampling 960 independent solutions for a single network instance with *N* = 10^5^ nodes and *M* = 5 × 10^5^ links.

From the algorithmic point of view, the local-consensus mechanism is very similar to a greedy algorithm which repeatedly selects among the whole network a highest-impact consumer node and changes it into a server^16^,^23^,^24^. Interestingly, we observe that the performance of the local-consensus mechanism slightly outperforms this greedy algorithm (Fig. 5). This surprising difference can be explained by two factors: first the local-consensus mechanism does not perform a global ranking of nodes, so a node of low impact value may become a server earlier than a node of much higher impact value; and second and more importantly, the local-consensus selection mechanism may convert a served consumer *i* to a server even if *i* has neighbors of higher impact values.

## Discussion

In this paper we considered the public service location problem as a cooperative game among *N* agents in a network, and presented a local-consensus selection mechanism through which a set of high-impact agents are appointed as hosts of service. We demonstrated that this decentralized selection mechanism can reduce the societal cost of service construction to a low level that is close to the lowest-possible value.

From the theoretical point of view, the demonstrated excellent performance of the local-consensus mechanism is very encouraging. Our work suggests that it is theoretically possible to efficiently solve the service location problem by distributed decision-making. The local-consensus mechanism does not need a central planner and it does not require the structural knowledge about the whole network. Furthermore, every agent participates in the decision-making process and its opinion has been incorporated in the final cooperative solution, which may help stabilizing the solution.

In this paper we considered only the construction cost of building the public services (e.g., schools and hospitals) and we proposed the local-consensus mechanism to minimize the total number of such facilities. Of course the operation cost of actually providing the service needs also be shared by the whole society. This latter issue might be easier to solve. For example, a simple cost-sharing mechanism might be that every consumer node contributes equally while every server gets reward proportional to the number of consumers it serves.

In determining the locations of the public services through the local-consensus mechanism, for simplicity we ignored the issue of possible future congestion in accessing service, but this is itself an interesting factor to explore^7^. We didn’t discuss the actual implementation of the local-consensus mechanism. Instead we assumed the ideal situation that every agent is cooperative and obeys the microscopic rules of the local-consensus mechanism. The practical feasibility of the local-consensus mechanism is an issue to be addressed in future empirical studies.

Collective problem-solving, division of labor, and role specialization are common not only in human societies but also in various other social systems such as social insects (e.g., ants and bees) and biological multi-cellular systems^25^,^26^,^27^ and swarms of robots^12^,^28^. For robotic systems, it might be relatively easy to implement the local-consensus decision-making mechanism to facilitate efficient division of labor and collective problem-solving.

## Additional Information

**How to cite this article**: Sun, Y.-F. and Zhou, H.-J. Serving by local consensus in the public service location game. *Sci. Rep.* **6**, 32502; doi: 10.1038/srep32502 (2016).

## Supplementary Material

Supplementary Information

## Figures and Tables

**Table 1:** Solving the public service location problem on eight real-world networks.

	*N*	*M*	*c*	BR	LS	LC	BPD
Facebook^30^	4039	88234	43.69	0.189	0.0649	0.00248	0.00248
PowerGrid^31^	4941	6594	3.265	0.486	0.353	0.3097	0.2997
CondMat^32^	23133	93439	8.078	0.357	0.239	0.1581	0.1558
Gnutella^33^	62586	147892	4.726	0.597	0.444	0.2018	0.201
LocGowalla^34^	196591	950327	9.668	0.478	0.382	0.2142	0.2119
DBLP^35^	317080	1049866	6.622	0.416	0.277	0.1471	0.1473
RoadNet-PA^36^	1088092	1541898	2.834	0.423	0.362	0.3335	0.3047
YouTube^35^	1134890	2987624	5.265	0.615	0.456	0.1881	0.1878

For each network, we list the number of nodes *N*, the number of links *M*, the mean degree *c* (2*M*/*N*), the mean fraction *n*_1_ of servers in the solutions obtained by four methods, namely the best-response (BR) mechanism, the local-share (LS) mechanism, the local-consensus (LC) mechanism, and the global BPD algorithm. Each *n*_1_ value in this table is the averaged result over 960 (for the first six network instances) or 48 (for the last two instances) independent simulations.
