# Supplementary material for: Serving by local consensus in the public service location game
Source: Sci Rep. 2016 Sep 2;6:32502. doi: 10.1038/srep32502 (PMC5009431; doi:10.1038/srep32502)
Supplement: Supplementary Information [file srep32502-s1.pdf]

Serving by local consensus in the public service location game

Yi-Fan Sun and Hai-Jun Zhou

Supplementary Information

Supplementary Figure 1

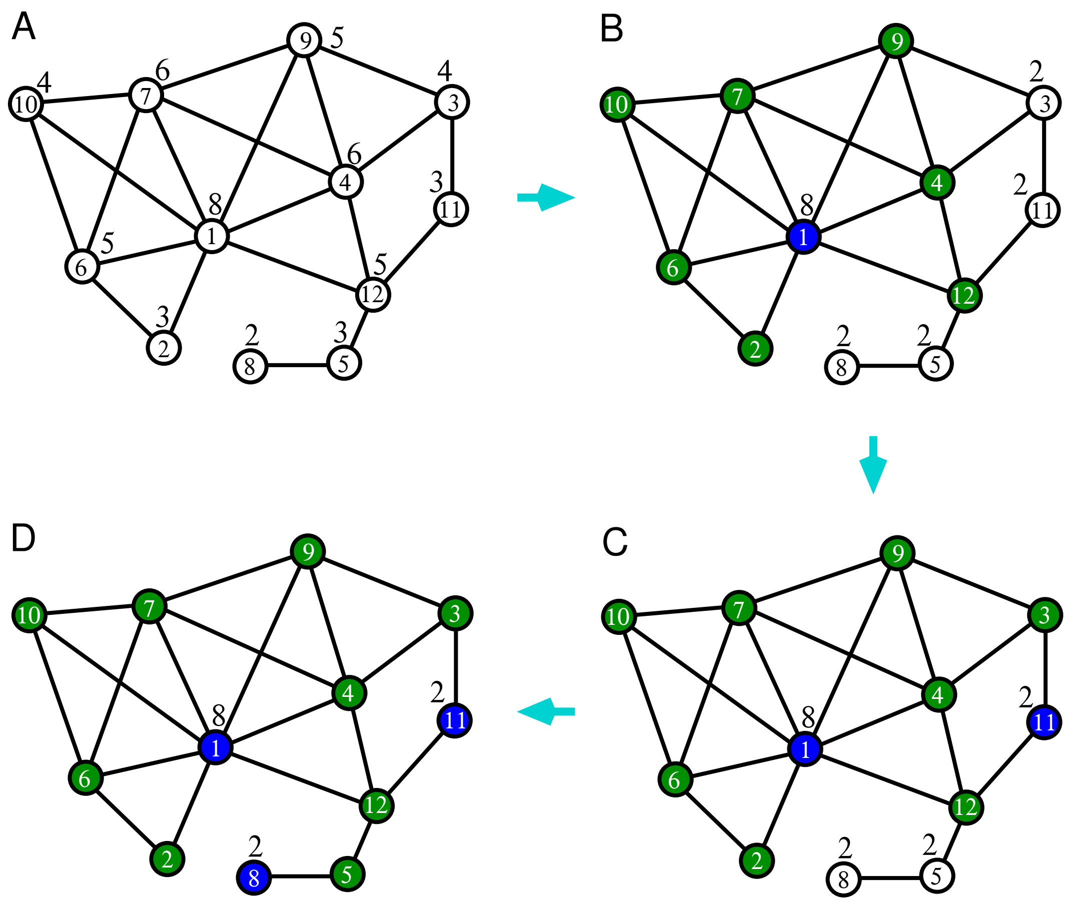

Figure S1. The local-share mechanism for solving the public service location problem. Blue nodes are servers, green nodes are served consumers, and white nodes are unserved consumers. The non-negative integer beside a node is its impact value. (A) Initially there is no server, and node 1 is the only candidate server who is agreed by all its neighbors. (B) Node 1 is chosen as a server, and every node in the set $\{1, 2, 4, 6, 7, 9, 10, 12\}$ pays an amount $c_1 = \frac{1}{8}$ for the service construction cost. (C) Node 11 is then chosen as a second server, and it and node 3 each pays an amount $c_2 = \frac{1}{2}$ for the construction cost. (D) Node 8 is then chosen as the third server, and it and node 5 each pays an amount $c_3 = \frac{1}{2}$ for the construction cost.

Supplementary Figure 2

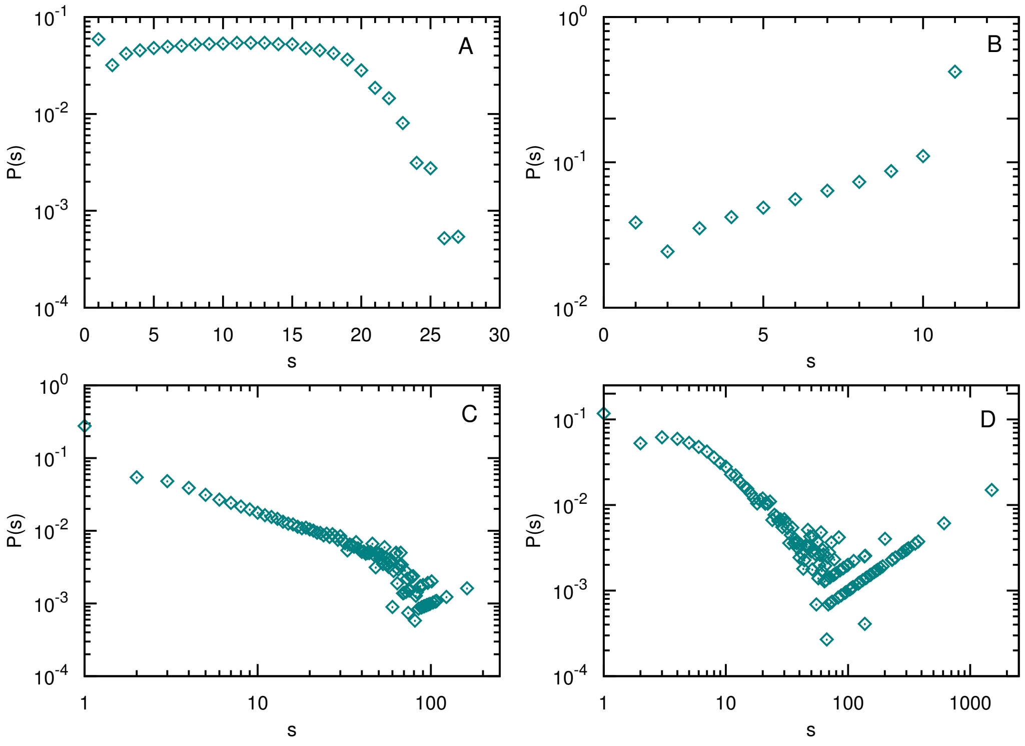

Figure S2. The local-share mechanism leads to inequality among different nodes of the network. According to this mechanism, if a group of s nodes share the unit construction cost of one public service, then each member in this group pays an equal amount of $\tau = \frac{1}{s}$. We show here the probability $P(s)$ that a randomly chosen node belongs to a sharing group of size s for four different network instances of the same size $N = 10^5$ and mean degree $c = 10$: (A) Erdös-Rényi, (B) regular random, (C) exponential, and (D) scale-free with decay exponent $\gamma = 3.0$. The size s has a wide distribution in all these four types of networks, and consequently the construction costs paid by different nodes differ considerably.

S1. MEAN FIELD THEORY FOR THE BEST RESPONSE DYNAMICS

Here we present a mean field theory to compute the final fraction n_1 of occupied (server) nodes under the best response dynamics. This theory is applicable for a generic random network with node degree distribution $P(d)$. It focuses on the evolution of the following quantities:

$\overline{N}_u(t)$: mean number of unserved nodes at time t;

$P_u(d;t)$: probability that at time t a randomly chosen unserved node has degree d;

$\overline{H}_1(t)$: mean value of the sum of degrees of all the occupied nodes at time t;

$\overline{H}_0(t)$: mean value of the sum of degrees of all the unoccupied nodes at time t;

$\overline{H}_u(t)$: mean value of the sum of degrees of all the unserved nodes at time t.

Initially all the nodes are unoccupied so $\overline{H}_0(0) = 2M = Nc$, where c is the mean node degree. At each time interval $\delta t \equiv \frac{1}{N}$ a randomly chosen unserved node is occupied, therefore

$$\overline{H}_0(t+\delta t) = \overline{H}_0(t) - \sum_d P_u(d;t) d \ . \tag{S1}$$

On the other hand, $\overline{H}_1(0) = 0$ and

$$\overline{H}_1(t+\delta t) = \overline{H}_1(t) + \sum_d P_u(d;t) d \ . \tag{S2}$$

The mean accumulated degree $\overline{H}_u(t)$ is expressed as

$$\overline{H}_u(t) = \overline{N}_u(t) \sum_d P_u(d;t) d \ . \tag{S3}$$

Initially all the nodes are unserved, $\overline{N}_u(0) = N$. At each time interval δt a randomly chosen unserved node is occupied and all the unserved neighbors of this newly occupied node become served. Since the nearest neighbors of each occupied node are all unoccupied, the probability at time t of a randomly chosen neighbor of an unserved node also being unserved is equal to $\frac{\overline{H}_u(t)}{\overline{H}_0(t) - \overline{H}_1(t)}$. Consequently the evolution of the number of unserved nodes is governed by

$$\overline{N}_u(t+\delta t) = \overline{N}_u(t) - 1 - \sum_d P_u(d;t) \frac{\overline{H}_u(t) d}{\overline{H}_0(t) - \overline{H}_1(t)} \ . \tag{S4}$$

From this equation we can obtain the evolution equation for $P_u(d;t)$ as

$$P_u(d;t+\delta t) = \frac{P_u(d;t)\Big[1 - \frac{1}{\overline{N}_u(t)} - \frac{\frac{\overline{H}_u(t)d}{\overline{H}_0(t)-\overline{H}_1(t)}}{\overline{N}_u(t)}\Big]}{1 - \frac{1}{\overline{N}_u(t)} - \frac{\frac{\overline{H}_u(t)}{\overline{H}_0(t)-\overline{H}_1(t)}\sum_{d'} P_u(d';t)d'}{\overline{N}_u(t)}} \ . \tag{S5}$$

Let us define several intensive quantities $h(t)$, $h_u(t)$, $\rho_u(t)$, and $c_u(t)$ as:

$$h(t) \equiv \big[\overline{H}_0(t) - \overline{H}_1(t)\big]/N \ , \tag{S6a}$$

$$h_u(t) \equiv \overline{H}_u(t)/N \ , \tag{S6b}$$

$$\rho_u(t) \equiv \overline{N}_u(t)/N \ , \tag{S6c}$$

$$c_u(t) \equiv \sum_d P_u(d;t)d \ . \tag{S6d}$$

$\rho_u(t)$ is the fraction of unserved nodes at time t, and $c_u(t)$ is the mean degree of unserved nodes at time t. From Eq. (S3) we know that

$$h_u(t) = \rho_u(t)c_u(t) \ . \tag{S7}$$

Furthermore, at the limit of $N \to \infty$ we have

$$\frac{\partial h(t)}{\partial t} = -2c_u(t) \ , \tag{S8}$$

$$\frac{\partial \rho_u(t)}{\partial t} = -1 - \frac{h_u(t)}{h(t)}c_u(t) \ , \tag{S9}$$

and the evolution of $P_u(d;t)$ is governed by

$$\frac{\partial P_u(d;t)}{\partial t} = P_u(d;t)\frac{h_u(t)}{\rho_u(t)h(t)}\big[c_u(t) - d\big] \ . \tag{S10}$$

Combining Eq. (S10) and Eq. (S7) we can obtain an explicit expression for $P_u(d;t)$ as

$$P_u(d;t) = \frac{P(d)\exp\big(-d\int_0^t \frac{c_u(t')}{h(t')}\mathrm{d}t'\big)}{\sum\limits_{d'} P(d')\exp\big(-d'\int_0^t \frac{c_u(t')}{h(t')}\mathrm{d}t'\big)} \ . \tag{S11}$$

Because of Eq. (S8) we know that

$$\int_0^t \frac{c_u(t')}{h(t')}\mathrm{d}t' = -\frac{1}{2}\ln\Big[\frac{h(t)}{c}\Big] \ . \tag{S12}$$

Plugging this expression into Eq. (S11) we finally obtain that

$$P_u(d;t) = \frac{P(d)\big[h(t)/c\big]^{d/2}}{\sum\limits_{d'} P(d')\big[h(t)/c\big]^{d'/2}} \ . \tag{S13}$$

while $h(t)$ is obtained by solving the self-consistent equation

$$\frac{\partial h(t)}{\partial t} = -2 \frac{\sum_{d} P(d) d \big[h(t)/c\big]^{d/2}}{\sum_{d'} P(d') \big[h(t)/c\big]^{d'/2}} \tag{S14}$$

under the initial condition of $h(0) = c$. Equation (S14) can be solved by numerical integration for a generic node degree distribution $P(d)$, while it can be solved analytically for some particular $P(d)$ forms (see below). With $h(t)$ known, we can then obtain $c_u(t)$ from Eq. (S8) and then apply Eq. (S9) to obtain $\rho_u(t)$ as the solution of the following differential equation

$$\frac{\partial \rho_u(t)}{\partial t} = -1 - \rho_u(t) \frac{\big[c_u(t)\big]^2}{h(t)} \ . \tag{S15}$$

As time t increases the fraction $\rho_u(t)$ of unserved nodes decreases continuously and approaches zero at certain threshold time t^*, i.e., $\rho_u(t^*) = 0$. Since a node is occupied at each time interval δt, the final fraction n_1 of occupied nodes during this best response dynamics is simply $n_1 = t^*$.

In the following subsections we apply this mean field theory to several simple network ensembles.

Erdös-Rényi network

The degree distribution for an ER network is

$$P(d) = \frac{e^{-c} c^d}{d!} \ , \tag{S16}$$

For this random network ensemble we have

$$h(t) = c(1-t)^2 \ , \tag{S17a}$$

$$c_u(t) = c(1-t) \ , \tag{S17b}$$

$$\rho_u(t) = \frac{1+c}{c} e^{-ct} - \frac{1}{c} \ . \tag{S17c}$$

Therefore the fraction of occupied nodes n_1 is

$$n_1 = \frac{\ln(1+c)}{c} \ . \tag{S18}$$

Equation (S18) was derived earlier in Ref. [6] following the probabilistic approach of Ref. [21].

Regular random network

In a regular random network every node has the same (integer) degree $c = K$, therefore

$$P(d) = \delta_d^K \ . \tag{S19}$$

For this random network ensemble we have

$$h(t) = K(1-2t) \ , \tag{S20a}$$

$$c_u(t) = K \ , \tag{S20b}$$

$$\rho_u(t) = \frac{K-1}{K-2}(1-2t)^{K/2} - \frac{1-2t}{K-2} \ . \tag{S20c}$$

Therefore the fraction of occupied nodes n_1 is

$$n_1 = \frac{1}{2}\big[1 - (K-1)^{\frac{2}{2-K}}\big] \ . \tag{S21}$$

Equation (S21) was derived earlier in Ref. [21].

Exponential random network

The degree distribution for an exponential random network of mean degree c is

$$P(d) = \frac{1}{1+c}\Big(\frac{c}{1+c}\Big)^d \ . \tag{S22}$$

For this random network ensemble we have

$$h(t) = \frac{(1+c-\sqrt{1+2ct})^2}{c} \ , \tag{S23a}$$

$$c_u(t) = \frac{1+c-\sqrt{1+2ct}}{\sqrt{1+2ct}} \ , \tag{S23b}$$

$$\rho_u(t) = \frac{1}{3c}\Big[\frac{1+3c}{\sqrt{1+2ct}} - (1+2ct)\Big] \ . \tag{S23c}$$

Therefore the fraction of occupied nodes n_1 is

$$n_1 = \frac{(1+3c)^{\frac{2}{3}} - 1}{2c} \ . \tag{S24}$$

The correctness of Eq. (S24) has been confirmed by our numerical simulation results.

S2. THE BPD ALGORITHM FOR THE SERVICE LOCATION PROBLEM

Selecting a minimum set of agents as service providers in a network is an intrinsically difficult combinatorial optimization problem. In the computer science literature this problem is usually referred to as the minimum dominating set problem. The spin glass model for the minimum dominating set problem and the associated replica-symmetric mean field theory have already been discussed in great detail [18]. Here we briefly review this mean field theory and the BPD message-passing algorithm.

Given an input network G, the marginal probability $q_i^{c_i}$ that a node i of this graph is in the occupation state $c_i \in \{0, 1\}$ is estimated by

$$q_i^{c_i} = \frac{e^{-x c_i} \prod_{j \in \partial i} \sum_{c_j} q_{j \to i}^{(c_j, c_i)} - \delta_0^{c_i} \prod_{j \in \partial i} q_{j \to i}^{(0,0)}}{\sum_{c_i'} e^{-x c_i'} \prod_{j \in \partial i} \sum_{c_j} q_{j \to i}^{(c_j, c_i')} - \prod_{j \in \partial i} q_{j \to i}^{(0,0)}} , \tag{S25}$$

where x is a positive re-weighting parameter; the Kronecker symbol $\delta_m^n = 1$ if $m = n$ and $\delta_m^n = 0$ if otherwise; and ∂i denotes the set of neighboring nodes of node i. For a link (i, j) between two nodes i and j, we denote by $q_{j \to i}^{(c_j, c_i)}$ the joint probability that i is in occupation state c_i and j is in occupation state c_j when the constraint of node i (that is, i should be occupied or be surrounded by at least one occupied neighbor) is not considered. This 'cavity' probability can be evaluated through the following belief-propagation (BP) equation:

$$q_{j \to i}^{(c_j, c_i)} = \frac{e^{-x c_j} \prod_{k \in \partial j \backslash i} \sum_{c_k} q_{k \to j}^{(c_k, c_j)} - \delta_0^{c_i + c_j} \prod_{k \in \partial j \backslash i} q_{k \to j}^{(0,0)}}{\sum_{c_i', c_j'} e^{-x c_j'} \prod_{k \in \partial j \backslash i} \sum_{c_k'} q_{k \to j}^{(c_k', c_j')} - \prod_{k \in \partial j \backslash i} q_{k \to j}^{(0,0)}} , \tag{S26}$$

where $\partial j \backslash i$ denotes the subset obtained by deleting node i from set ∂j.

Equations (S25) and (S26) are exploited by the BPD algorithm to construct a near-optimal dominating set for the network G. The details of the BPD algorithm are given in [18]. Roughly speaking, at each round of the BPD process, first the BP equation (S26) is iterated on the network a few number of times, then the occupation probabilities $q_i^{c_i}$ of all the unoccupied nodes i are estimated by Eq. (S25), and then those nodes with the highest probabilities of being occupied are set to be occupied. More and more nodes become occupied as the BPD process continues, and it stops as soon as a dominating set is reached.

The sizes of dominating sets constructed by the BPD algorithm are not sensitive to the re-weighting parameter x. We fix the value of x to be $x = 10$ in the present work.

S3. TIME COMPLEXITY OF THE FOUR ALGORITHMS

The BPD algorithm is a message-passing algorithm. For a sparse network with each node only connects to a small fraction of all the other nodes, the algorithm takes a time proportional to $N \ln(N)$ to reach a dominating set solution [18], where N is the total number of nodes in the network.

All the three decentralized local mechanisms (best-response, local-share, local-consensus) have linear time complexity to construct a solution for the public service location problem.
